# Detection, Location, and Classification of Multiple Dipole-like Magnetic Sources Based on L2 Norm of the Vertical Magnetic Gradient Tensor Data

**DOI:** 10.3390/s23094440

**Published:** 2023-05-01

**Authors:** Lin Ge, Qi Han, Xiaojun Tong, Yizhen Wang

**Affiliations:** School of Computer Science and Technology, Harbin Institute of Technology, Harbin 150001, China; 18b903071@stu.hit.edu.cn (L.G.); tong_xiaojun@163.com (X.T.); yizhen@stu.hit.edu.cn (Y.W.)

**Keywords:** unknown dipole quantity, multiple magnetic dipole detection, magnetic gradient tensor, nonlinear optimization, normalized source strength

## Abstract

In recent years, there has been a growing interest in the detection, location, and classification (DLC) of multiple dipole-like magnetic sources based on magnetic gradient tensor (MGT) data. In these applications, the tilt angle is usually used to detect the number of sources. We found that the tilt angle is only suitable for the scenario where the positive and negative signs of the magnetic sources’ inclination are the same. Therefore, we map the L2 norm of the vertical magnetic gradient tensor on the arctan function, denoted as the VMGT${}_{2}$ angle, to detect the number of sources. Then we use the normalized source strength (NSS) to narrow the parameters’ search space and combine the differential evolution (DE) algorithm with the Levenberg–Marquardt (LM) algorithm to solve the sources’ locations and magnetic moments. Simulation experiments and a field demonstration show that the VMGT${}_{2}$ angle is insensitive to the sign of inclination and more accurate in detecting the number of magnetic sources than the tilt angle. Meanwhile, our method can quickly locate and classify magnetic sources with high precision.

## 1. Introduction

Detection, location, and classification (DLC) of multiple magnetic sources has important applications in unexploded ordnance detection [1], exploration of mineral resources [2], magnetic characterization of scientific spacecraft [3], biological medical engineering [4,5], and other fields. Since the magnetic fields generated by multiple magnetic sources overlap and vary nonlinearly with the distance, the DLC of multiple magnetic sources is challenging. Usually, the shape and size of the magnetic source are different. When the distance between the magnetic source and the measurement point is 2.5 times larger than the source length, the magnetic source can be regarded as a dipole-like magnetic source [6]. A single dipole-like magnetic source contains six parameters to describe its position information (x,y,z) and magnetic moment information $\left(M_{x},M_{y},M_{z}\right)$. Therefore, the DLC of multiple magnetic sources can be transformed into the problem of detecting the number and solving parameters of multiple dipole-like magnetic sources.

Several methods have been proposed for the DLC of magnetic sources. Yousefi et al. [7] used the Levenberg–Marquardt (LM) algorithm to locate and classify magnetic objects. Carrubba et al. [8] considered that the minimum value of the objective function corresponds to the correct number of dipole-like sources, so he searched for the quantity, location, and magnetic moment best matching the measured field by the particle swarm optimization (PSO) algorithm. However, this method is ill-posed and easily falls into a locally optimal solution. The magnetic gradient tensor (MGT) data contain more information about the magnetic sources, which can reduce the interference of the geomagnetic diurnal variation and the regional magnetic fields [9,10,11]. With the maturity of measured MGT data [12], there hass been more and more research on the DLC of multi-magnetic sources based on MGT data. Chang et al. [13] added a parameter indicating the truth or falsity of the magnetic sources and optimized it jointly with other dipole parameters by the LM algorithm. This method increases the dimension of the parameter space, making it challenging to meet the engineering requirements with the DLC of multiple magnetic sources. Gang et al. [14] first used the tilt angle to detect the number of magnetic sources. They used a stepwise method to locate and classify magnetic sources based on MGT data. Ding et al. [15] improved the tilt angle and combined the stepwise method with the differential evolution (DE) algorithm to solve the parameters of the magnetic source. The tilt angle was proposed by Miller [16] for locating the potential field sources, which is applied under the premise that its value is positive above the potential field sources. However, this premise is invalid for the magnetic field. Based on the definition of the tilt angle, it is positive or negative depending on the magnetic inclination. If multiple magnetic sources with different positive and negative inclinations are in the detection area simultaneously, the estimated number of magnetic sources may have a high probability of error. Recently, Li et al. [17] improved the tilt angle based on the normalized source strength (NSS) and the contraction tensor (CT). They used a threshold method to estimate the sources’ numbers. However, some outliers of the tilt angle exist between sources, which affects the correctness of the estimated number of magnetic sources. Setting a reasonable threshold is also challenging, requiring prior information about the magnetic source.

All of the methods above for solving the magnetic source parameters are either sensitive to the setting of the initial value of the parameters or have a slow optimization convergence speed. In the field of magnetic target tracking, in order to track the target quickly and accurately, the PSO-LM hybrid algorithm has been proposed and widely applied [18,19,20]. This algorithm uses the PSO algorithm to iteratively optimize toward an approximate global optimal solution and then uses this approximate solution as an initial parameter and executes the LM algorithm. This hybrid method can improve the convergence speed while ensuring correctness, thus effectively avoiding the shortcomings mentioned above. In the DLC of multiple magnetic sources, when faced with a large number of magnetic sources, the PSO-LM algorithm has poor convergence due to the optimization ability of the PSO algorithm [21]. The DE algorithm has been proved to be able to converge to the optimal solution with high precision [15], but the convergence speed is slow, especially near the global optimal solution.

**Figure 1:**
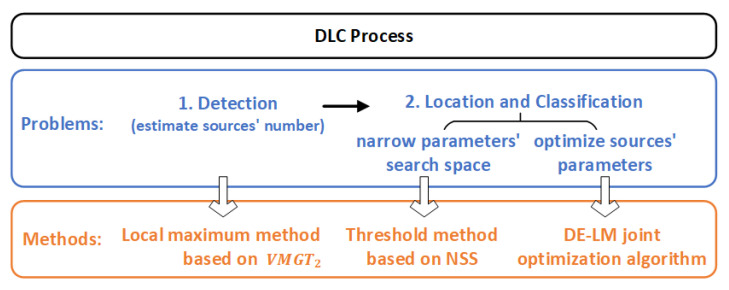
Process of DLC of multiple magnetic sources.

In order to improve the correctness of detecting the sources’ number and quickly converging the parameters to a high-precision global optimal solution, we first calculate the local maximum of the VMGT${}_{2}$ angle to estimate the number of sources. Then, we use the NSS to narrow the search space of the horizontal position parameters of the magnetic source and combine the DE and LM algorithms to optimize the parameters of the magnetic source. The overall process of our methods is shown in Figure 1.

This paper is organized as follows. In Section 2, a detailed description of our methods is presented. Section 3 validates the proposed algorithm through simulation and real experiments. Finally, Section 4 concludes this paper with some remarks and future research directions.

## 2. Analysis and Methods

### 2.1. MGT and the Tilt Angle

**Figure 2:**
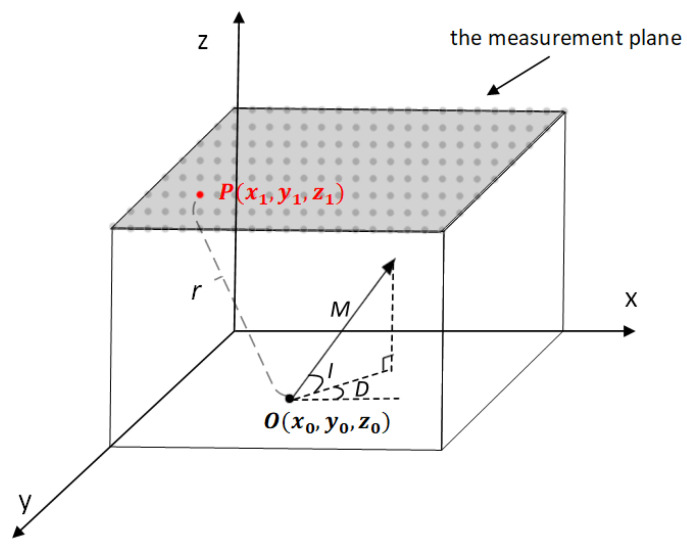
Coordinate system for magnet’s DLC.

Assuming that a magnetic dipole is located at point $O(x_{0},y_{0},z_{0})$ in the Cartesian coordinate system shown in Figure 2, the magnetic field B generated at point $P(x_{1},y_{1},z_{1})$ can be expressed as:

(1) $$B=\frac{\mu }{4\pi r^{5}}\left[\begin{array}{ccc} 3x^{2}-r^{2} & 3xy & 3xz\\ 3xy & 3y^{2}-r^{2} & 3yz\\ 3xz & 3yz & 3z^{2}-r^{2} \end{array}\right]\left[\begin{array}{c} m_{x}\\ m_{y}\\ m_{z} \end{array}\right]$$

where μ is the magnetic permeability of the free space, *r* is the distance between the magnetic dipole and the measurement point, $x=x_{1}-x_{0}$, $y=y_{1}-y_{0}$, $z=z_{1}-z_{0}$. $m_{x}$, $m_{y}$, and $m_{z}$ are the components of the magnetic dipole moment in the three axes, respectively, which can be further expressed as $m_{x}=M\cdot cosI\cdot cosD$, $m_{y}=M\cdot cosI\cdot sinD$, $m_{z}=M\cdot sinI$. *I* is the magnetic inclination in the range of $\left[-90^{\circ },90^{\circ }\right]$, *D* is the magnetic deflection in the range of $\left[-180^{\circ },180^{\circ }\right]$, and *M* is the magnetic moment constant.

The MGT is the second-order tensor of the total magnetic field, containing a total of nine components and can form a 3 ×3 matrix. According to the Maxwell equation, in a passive static magnetic field, the divergence and curl of the magnetic field are equal to zero, that is, $B_{xy}=B_{yx}$, $B_{xz}=B_{zx}$, $B_{yz}=B_{zy}$, $B_{xx}+B_{yy}+B_{zz}=0$. Hence, a single measurement point can provide five independent components of MGT, expressed as

(2) $$G=\left[\begin{array}{c} B_{xx}\\ B_{xy}\\ B_{xz}\\ B_{yy}\\ B_{yz} \end{array}\right]=\frac{3\mu }{4\pi r^{7}}\left[\begin{array}{ccc} 3xr^{2}-5x^{3} & yr^{2}-5x^{2}y & zr^{2}-5x^{2}z\\ yr^{2}-5x^{2}y & xr^{2}-5xy^{2} & -5xyz\\ zr^{2}-5x^{2}z & -5xyz & xr^{2}-5xz^{2}\\ xr^{2}-5xy^{2} & 3yr^{2}-5y^{3} & zr^{2}-5y^{2}z\\ -5xyz & zr^{2}-5y^{2}z & yr^{2}-5yz^{2} \end{array}\right]\left[\begin{array}{c} m_{x}\\ m_{y}\\ m_{z} \end{array}\right]$$

The invariant *c* is the CT and the invariant *u* is the NSS. They can be calculated by the tensor matrix **G** and do not change with the rotation of the coordinate system. $\lambda_{1}$, $\lambda_{2}$, and $\lambda_{3}$ are the eigenvalues of G.

(3) $$\begin{array}{cc} c & =\sqrt{B_{xx}^{2}+B_{yy}^{2}+B_{zz}^{2}+2\left(B_{xy}^{2}+B_{yz}^{2}+B_{xz}^{2}\right)} \end{array}$$

(4) $$\begin{array}{cc} u & =\sqrt{-\lambda_{3}^{2}-\lambda_{1}\lambda_{2}} \end{array}$$

The tilt angle is defined as the ratio of the first-order vertical derivative of the potential field to its horizontal gradient, where Tilt1−Tilt3 are different forms of tilt angles obtained based on the definition in  [15]. $T_{N}$ and $T_{C}$ are two improved tilt angles generated by NSS and CT, respectively [17].

(5) $$\begin{array}{cc} Tilt1 & ={tan}^{-1}\left(B_{zz}/\sqrt{{B_{xx}}^{2}+{B_{yy}}^{2}}\right) \end{array}$$

(6) $$\begin{array}{cc} Tilt2 & ={tan}^{-1}\left(B_{zz}/\sqrt{{B_{zx}}^{2}+{B_{zy}}^{2}}\right) \end{array}$$

(7) $$\begin{array}{cc} Tilt3 & ={tan}^{-1}\left(B_{z}/\sqrt{{B_{x}}^{2}+{B_{y}}^{2}}\right) \end{array}$$

(8) $$\begin{array}{cc} T_{N} & =sgn\left(\frac{\partial u}{\partial z}\right){tan}^{-1}\left(\frac{\partial u/\partial z}{\sqrt{\left({\partial u/\partial x}^{2}+{\partial u/\partial y}^{2}+{\partial u/\partial z}^{2}\right)}}\right) \end{array}$$

(9) $$\begin{array}{cc} T_{C} & =sgn\left(\frac{\partial c}{\partial z}\right){tan}^{-1}\left(\frac{\partial c/\partial z}{\sqrt{\left({\partial c/\partial x}^{2}+{\partial c/\partial y}^{2}+{\partial c/\partial z}^{2}\right)}}\right) \end{array}$$

where sgn(.) is the sign function; ∂u/∂x, ∂u/∂y, ∂c/∂x, and ∂c/∂y are the horizontal derivatives of the invariants; ∂u/∂z and ∂c/∂z are the vertical derivatives obtained by the Fourier transform in the frequency domain.

### 2.2. Estimation of the Number of Magnetic Sources Based on the VMGT${}_{2}$ Angle

Since multiple dipole-like magnetic sources may have different magnetic moments and distribute at different depths, the magnetic vector and MGT generated by different sources may have different orders of magnitude at a measurement point. Therefore, it is impossible to estimate the number of magnetic sources directly using the magnetic vector or MGT data. The previous analysis shows that the tilt angle is unsuitable for scenarios where the magnetic sources have different positive and negative inclinations simultaneously.

Our approach is to map the L2 norm of the vertical gradient tensor on the arctan trigonometric function, denoted as the VMGT${}_{2}$ angle, to estimate the magnetic sources’ number, as shown in Equation (10). Based on the characteristic that the MGT data fall off as $1/r^{4}$, where r is the distance from the magnetic source, there is little mutual interference of the VMGT${}_{2}$ angle between different magnetic sources, and the VMGT${}_{2}$ angle is much larger in the vicinity of the magnetic source than in other areas. Moreover, the VMGT${}_{2}$ angle can detect sources with a small inclination by introducing the gradient tensors $B_{xz}$ and $B_{yz}$. By calculating the L2 norm of the gradient tensor, the VMGT${}_{2}$ angle is insensitive to the positive and negative inclination. Finally, mapping to the arctan trigonometric function can limit the VMGT${}_{2}$ angle between $0^{\circ }$ and $90^{\circ }$, which can reduce the impact that various magnetic moments and depths have on the detection of the magnetic source.

(10) $$\begin{array}{c} f={tan}^{-1}\left(\sqrt{{B_{zx}}^{2}+{B_{zy}}^{2}+{B_{zz}}^{2}}\right) \end{array}$$

We calculate the local maximum points of the VMGT${}_{2}$ angle on the measurement plane and use its number as the estimated number of magnetic sources. The pseudo-code is presented in Algorithm 1. We assume that P(i,j) is the horizon position of the measurement point, and f(i,j) is the VMGT${}_{2}$ angle at P(i,j) calculated by Equation (10). Therefore, the horizontal gradients $f_{x}$ and $f_{y}$ of the VMGT${}_{2}$ angle can be calculated as:

(11) $$\begin{array}{ll} \; & f_{x}\left(i,j\right)=\left\{\begin{array}{cc} {}[f(i,j+1)-f(i,j-1)]/2 & \mathrm{if}\;(1<j<end)\\ {}[f(i,j+1)-f(i,j)]/2 & \mathrm{if}\;(j=1)\\ {}[f(i,j)-f(i,j-1)]/2 & \mathrm{if}\;(j=end) \end{array}\right. \end{array}$$

(12) $$\begin{array}{ll} \; & f_{y}\left(i,j\right)=\left\{\begin{array}{cc} {}[f(i+1,j)-f(i-1,j)]/2 & \mathrm{if}\;(1<i<end)\\ {}[f(i+1,j)-f(i,j)]/2 & \mathrm{if}\;(i=1)\\ {}[f(i,j)-f(i-1,j)]/2 & \mathrm{if}\;(i=end) \end{array}\right. \end{array}$$

The calculation formula of the local maximum point $S(i_{source},j_{source})$ is:

(13) $$\begin{array}{ll} j_{source} & =\left\{\begin{array}{cc} j & \mathrm{if}\;(\;|f_{x}\left(i,j\right)|\;\leq \;|f_{x}\left(i,j+1\right)|\;)\\ j+1 & \mathrm{otherwise}. \end{array}\right. \end{array}$$

(14) $$\begin{array}{ll} i_{source} & =\left\{\begin{array}{cc} i & \mathrm{if}\;(\;|f_{y}\left(i,j_{source}\right)|\;\leq \;|f_{y}\left(i+1,j_{source}\right)|\;)\\ i+1 & \mathrm{otherwise}. \end{array}\right. \end{array}$$

**Algorithm 1** Estimate Sources’ Number Based on the VMGT${}_{2}$ angle.**Input**   : MGT data G;**Output** : Magnetic sources’ number N;**Procedure** :***Step 1***: Calculate the VMGT${}_{2}$ angle of every measurement point through Equation (10).***Step 2***: Calculate $f_{x}\left(i,j\right)$ and $f_{y}\left(i,j\right)$ through Equations (11) and (12).***Step 3***: Find measurement points that satisfy the condition $f_{x}\left(i,j\right)>0$ and $f_{x}\left(i,j+1\right)\leq 0\;$, and calculate $j_{source}$ through Equation (13).***Step 4***: Find measurement points that satisfy the condition $f_{y}\left(i,j_{source}\right)>0$ and $f_{y}\left(i+1,j_{source}\right)\leq 0$, and calculate $i_{source}$ through Equation (14).***Step 5***: calculate the number of $S_{p}\left(i_{source},j_{source}\right)$ and denote it as N.

### 2.3. Solving Dipole-like Magnetic Source Parameters

#### 2.3.1. Narrow Parameters’ Search Space Based on NSS

The NSS is isotropic around the magnetic dipole and independent of the magnetization direction [16,22]. Since NSS falls off as $1/r^{4}$, there is less interference between each magnetic source. The increasing values of NSS unambiguously indicate a closer approach to a target [14]. So we take the local maximum point of NSS as the approximate horizontal position of the magnetic source. It is worth noting that the approximate horizontal position cannot be used directly as the horizontal parameters of magnetic sources when a magnetic source lies between measurement points. However, based on the approximate horizontal position, we can narrow the search space of the horizontal parameter of the magnetic source, which will effectively prevent the algorithm from falling into a local optimal solution.

The local maximum point of NSS exists not only above the magnetic source; it may also exist between different dipoles [14], which will interfere with the solution of the dipoles’ approximate horizontal position. Therefore, we set a threshold σ to divide the detection area into multiple anomaly areas based on the VMGT${}_{2}$ angle. Then, we calculate the local maxima points $\left(x_{0},y_{0}\right)$ of NSS in each anomaly area. The initial value of σ is set to the minimum value of the $VMGT_{2}$ angle in the area. It gradually increases with the number of divisions until each divided anomaly area contains only one point from $S_{p}$. Based on the local maxima points of the NSS, we narrow the search spaces of the horizontal position parameters as $\left[x_{0}-2\;l,x_{0}+2\;l\right]$ and $\left[y_{0}-2\;l,y_{0}+2\;l\right]$, where *l* is the interval of the measurement points.

#### 2.3.2. Optimize the Magnetic Source Parameters Using DE-LM Algorithm

The DE algorithm is a random search heuristic algorithm based on population differences. It was proposed by Storn and Price [23] to deal with global optimization problems. Its algorithm principle is similar to the genetic algorithm, including four processes: initialization, mutation, crossover, and selection. The characteristics of this algorithm are mainly reflected in the mutation process. Based on the difference strategy, the difference between individuals in the population is used to realize the variety of individuals. This type of algorithm is not sensitive to the setting of the initial value, so it usually adopts a strategy of random initialization. The DE algorithm can optimize multiple dipole parameters with high accuracy but requires more convergence time, especially near the global optimal solution.

The LM algorithm is a widely used non-linear least squares algorithm, which combines the characteristics of the gradient descent algorithm and the Gauss–Newton algorithm. The gradient descent method is used to determine the direction of the search, and the step length of the search is determined by the Gauss–Newton method. The LM algorithm can quickly converge to the optimal solution, but this algorithm is more sensitive to the initial values of the parameters. Unreasonable initial values may cause the algorithm to converge to a local optimum solution.

Here, we combine the DE algorithm [15] with the LM algorithm [7] to overcome these shortcomings. We iterate the DE algorithm to obtain an approximate global optimal solution close to the real value of the parameters. Then, we utilize the approximate solution as the initial parameter values of the LM algorithm and execute it. The LM algorithm can quickly converge to the global optimal solution that meets the accuracy requirements. The execution flow of the DE-LM algorithm is shown in Algorithm 2. It is worth mentioning that there are many strategies for the DE algorithm to stop, such as setting a fixed number of iterations, setting the threshold of the cost function value, or setting the threshold of the variation of the cost function value within the interval. To ensure the DE algorithm can converge to an approximate global optimal solution without iterating too many times, this paper adopts the strategy of stopping if the variation of the cost function value in the set interval is less than a certain threshold. Based on experience, we set the interval size to 50 iterations and the threshold to 0. That is, if the variation of the cost function within 50 iterations is 0, the DE algorithm is stopped.

Assuming that the DE-LM algorithm searches for the global optimal solution in *D*-dimensional real parameter space. $\vec{X_{i}^{g}}$ is used as a set of candidate solutions of the optimization problem in the *g*th generation to form a population $X^{g}$ of size *P*, which can be expressed as

(15) $$\begin{array}{c} \vec{X_{i}^{g}}=\left[X_{i,1}^{g},X_{i,2}^{g},\ldots ,X_{i,N}^{g}\right],\;\;\;i=1,2,\ldots ,P \end{array}$$

where *N* is the number of magnetic sources, $X_{i,n}^{g}=\left[x_{i,n}^{g},y_{i,n}^{g},z_{i,n}^{g},I_{i,n}^{g},D_{i,n}^{g},M_{i,n}^{g}\right]$ is the parameters of the *n*th magnetic source, n∈[1,N].

(16) $$\begin{array}{c} {X^{g}}^{*}=\underset{X^{g}}{argmin}\sum_{k=1}^{K}\left[G_{k}-g_{k}\left(\vec{X_{i}^{g}}\right)\right]{\left[G_{k}-g_{k}\left(\vec{X_{i}^{g}}\right)\right]}^{T} \end{array}$$

The set of magnetic source parameters ${X^{g}}^{*}$ represents the solution with the smallest mean square error sum at all measurement points between the measured value G and the estimated value $g({\vec{X}}_{i}^{g})$; *K* is the number of measurement points. The initialization strategy of the parameters is

(17) $$\begin{array}{c} X_{i,n,j}^{g}=X_{i,n,j}^{min}+rand_{i,j}\left(0,1\right)\times \left(X_{i,n,j}^{max}-X_{i,n,j}^{min}\right) \end{array}$$

where $X_{i,n,j}^{g}$ is the *j*th component of $X_{i,n}^{g}$, $\left[X_{i,n,j}^{min},X_{i,n,j}^{max}\right]$ is the search space of $X_{n,j}$, and $rand_{i,j}\left(0,1\right)$ is a random number between 0 and 1.
**Algorithm 2** DE-LM**Input**  : MGT data G;              Magnetic sources’ number N;              Parameters’ range R.**Output** : The Global Optimal Solution X*.**Procedure** :***Step 1***: Initialize the parameters through Equation (17).***Step 2***: Optimize the magnetic source parameters to an approximate global optimal solution X′ by DE algorithm  [15].***Step 3***: Set X′ as the initial value of the LM algorithm [7].***Step 4***: Execute the LM algorithm until the stop condition is satisfied.

## 3. Experiment

In this section, we set up two synthetic examples to simulate the DLC in the face of common and extreme numbers of magnetic sources in engineering. Then, we set up a simulation experiment to verify the detection capability of our method in the face of interfering magnetic sources. Finally, the applicability in engineering of our methods is verified by a field demonstration.

### 3.1. Evaluation Metrics

In order to evaluate the performance of DeLC of multiple magnetic dipole-like sources, we use the position error function $E_{p}$ and the orientation error function $E_{o}$ to evaluate the optimization accuracy [7]. The convergence time ratio $R_{t}$ is used to compare the convergence speed of different algorithms.

(18) $$\begin{array}{cc} E_{p} & =\frac{1}{N}\sum_{i=1}^{N}\sqrt{{\left(x^{ip}-x^{ie}\right)}^{2}+{\left(y^{ip}-y^{ie}\right)}^{2}+{\left(z^{ip}-z^{ie}\right)}^{2}} \end{array}$$

(19) $$\begin{array}{cc} E_{o} & =\frac{1}{N}\sum_{i=1}^{N}\sqrt{{\left(m_{x}^{ip}-m_{x}^{ie}\right)}^{2}+{\left(m_{y}^{ip}-m_{y}^{ie}\right)}^{2}+{\left(m_{z}^{ip}-m_{z}^{ie}\right)}^{2}} \end{array}$$

(20) $$\begin{array}{cc} R_{t} & =\frac{t_{algorithm1}}{t_{algorithm2}} \end{array}$$

where $\left(x^{ip},y^{ip},z^{ip},m_{x}^{ip},m_{y}^{ip},m_{z}^{ip}\right)$ is the real position and moment of the *i*th magnetic source. $\left(x^{ie},y^{ie},z^{ie},m_{x}^{ie},m_{y}^{ie},m_{z}^{ie}\right)$ is the estimated position and moment of the *i*th magnetic source. $t_{algorithm1}$ and $t_{algorithm2}$ represent the time that two algorithms take to converge to the global optimal solution.

### 3.2. Simulation Experiment

#### 3.2.1. DLC of a Common Number of Magnetic Sources

We simulated the DLC of a common number of magnetic sources in engineering to compare the correctness of our method with the tilt angle method in estimating the number of magnetic sources and to compare the parameter optimization effect with other algorithms. We set up five magnetic sources with different parameters, as shown in Table 1. In order to validate the method’s correctness for estimating the number of sources with different positive and negative inclinations, we set the inclinations of dipoles 2 and 4 as negative and set the inclinations of dipoles 1, 3, and 5 as positive. The detection area was 20 m × 20 m, the height of the measurement plane was 0 m, and the distance between the measurement points was 0.25 m.

**Figure 3:**
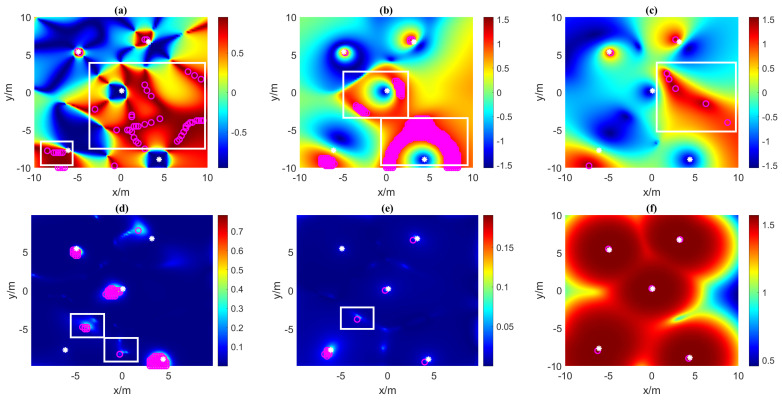
The tilt angle and VMGT${}_{2}$ angle on the measured plane. (**a**) Tilt1; (**b**) Tilt2; (**c**) Tilt3; (**d**) $T_{N}$; (**e**) $T_{C}$; (**f**) the VMGT${}_{2}$ angle. The white asterisks represent the real magnetic sources, the rose-red circles represent the detected magnetic sources, and the white rectangles represent the spurious magnetic anomaly regions.

To compare the correctness of the VMGT${}_{2}$ angle and the tilt angle for estimating the number of sources, we calculated Tilt1, Tilt2, Tilt3, $T_{C}$, $T_{N}$, and the VMGT${}_{2}$ angle on the measurement plane and plotted it in Figure 3. The white asterisks indicates the location of the real magnetic source. The rose-red circles represent the detected magnetic sources. In Figure 3a,b,d,e, the detected sources are obtained by the threshold method (threshold is 0.955, 1.2, 0.33, and 0.075, respectively). The threshold setting is the minimum value to ensure that every real magnetic source can be detected. In Figure 3c,f, the detected sources are the local maximum points. In Figure 3a–c, when the inclination is negative (such as dipole 2 and 4), the tilt angle directly above the magnetic source is negative, and the tilt angle around the area is positive. In this case, the magnetic source interacts with each other and generates some spurious regions marked by white rectangles. In Figure 3d,e, the value of $T_{C}$ and $T_{N}$ is always positive, but some outliers of the tilt angle also exist between sources, as shown in the white rectangles. The VMGT${}_{2}$ angle is insensitive to positive and negative inclinations and is less affected by other sources. It correctly estimates the number of magnetic sources, as shown in Figure 3f.

**Figure 4:**
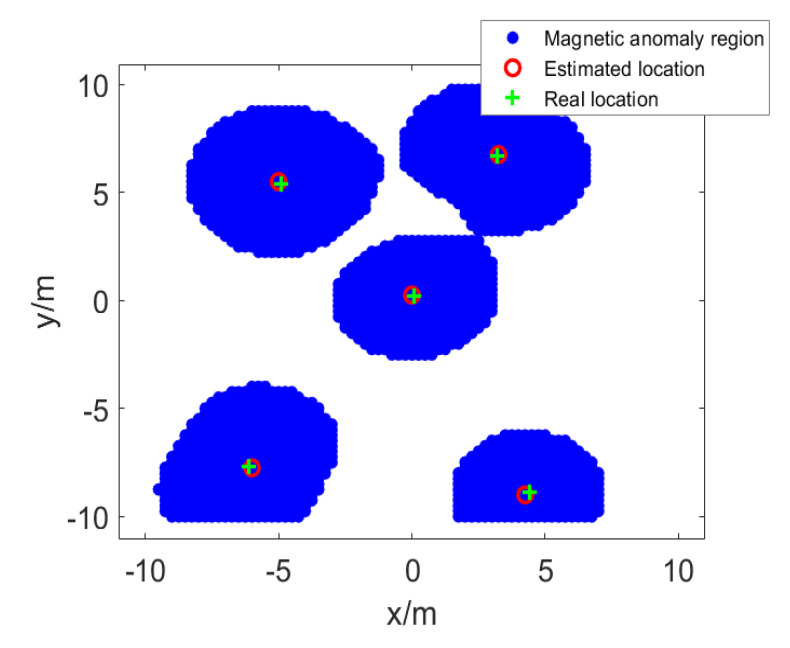
The magnetic anomaly region and the NSS.

Then, we use NSS to reduce the search space of the parameters of the horizontal position of the magnetic source. We set the initial value of the threshold σ as the minimum of the VMGT${}_{2}$ angle (σ = 0.514) in the detection area, and increase σ iteratively until each magnetic anomaly area only contains a single magnetic source. The divided magnetic anomaly areas and NSS are shown in Figure 4, where the blue area is the divided magnetic anomaly area, the green cross indicates the real position of the magnetic source, and the red circle indicates the position of the local maximum point of NSS in each magnetic anomaly area. Based on the local maximum point of NSS, we could obtain the reduced search space of the magnetic source parameters and record it in Table 2.

Finally, based on the estimated number of magnetic sources and the reduced search space of magnetic source parameters, we optimize the parameters of magnetic sources. We compared the optimization accuracy and convergence speed of the PSO-LM [20], DE [15], and DE-LM algorithms under different levels of Gaussian noise. We recorded the optimal solution and error of the parameters in Table 3. For noise-free cases, the DE algorithm and the DE-LM algorithm converged to a high accuracy solution $(E_{p}<1$ mm, $E_{o}<10^{-3}\;Am^{2})$; at this time, $R_{t}=t_{DE}/t_{DE-LM}=3.0301$, which means the convergence speed of the DE-LM algorithm is 3.0301 times faster than the DE algorithm. The PSO-LM algorithm converges to a locally optimal solution, which shows that the convergence of the PSO-LM algorithm is poor when there are many magnetic source parameters. For cases of 10% Gaussian random noises, the parameter’s optimization accuracy of the DE algorithm is $E_{p}=11$ mm and $E_{o}=0.146\;Am^{2}$, and the parameter’s optimization accuracy of the DE-LM algorithm is $E_{p}=10$ mm and $E_{o}=0.201\;Am^{2}$. The parameter optimization accuracy of both algorithms was similar. $R_{t}=t_{DE}/t_{DE-LM}=7.105$ means the DE-LM algorithm’s convergence speed was 7.105 times faster than that of the DE algorithm.

#### 3.2.2. DLC of an Extreme Number of Magnetic Dipoles

To simulate the DLC of an extreme number of magnetic sources in engineering to verify the applicability of our methods, we designed a synthetic example with 40 magnetic dipoles, as shown in Table 4. The survey area is 50 m × 50 m and the interval between the measurement points is 0.625 m.

**Figure 5:**
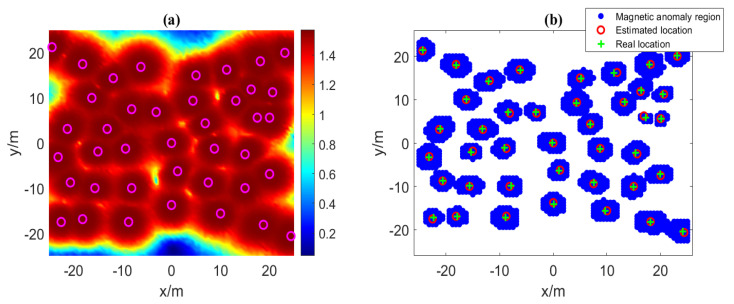
(**a**) Calculated VMGT${}_{2}$ angle and (**b**) NSS of 40 magnetic dipoles under 10% random Gaussian noise. The rose-red circles represent the detected magnetic sources.

Figure 5a shows the VMGT${}_{2}$ angle on the measurement plane under 10% random Gaussian noise. The rose-red circles are the detected sources obtained by the VMGT${}_{2}$ angle’s local maxima points. Figure 5b shows the NSS on the measurement plane under 10% random Gaussian noise. Obviously, our method correctly estimates the number of magnetic sources and obtains the approximate horizontal position of the magnetic source based on the NSS data.

The error of parameters estimated by the DE-LM algorithm is recorded in Table 5. For noise-free cases, the position error $E_{p}<1$ mm and the orientation error $E_{o}<10^{-3}\;Am^{2}$. For 10% Gaussian random noise cases, the position error was $E_{p}=24$ mm and the orientation error was $E_{o}=0.569\;Am^{2}$.

**Figure 6:**
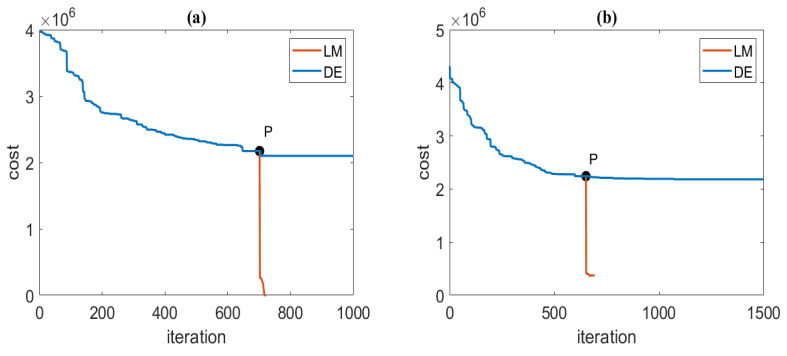
Cost function at each iteration of the DE-LM algorithm. (**a**) Noise-free; (**b**) 10% Gaussian random noise.

The iteration process of the DE-LM algorithm under noise-free and 10% Gaussian random noise are shown in Figure 6. The optimization process of the DE algorithm is represented by the blue line, and the red line represents the optimization processes of the LM algorithm. The convergence speed of the DE algorithm is slow after point P. We use the approximate optimal solution obtained by the DE algorithm at point P as the initial value of the LM algorithm and execute the LM algorithm. Compared with the DE algorithm, the convergence speed was effectively improved by combining it with the LM algorithm.

#### 3.2.3. Detection of Magnetic Sources Interfering with Each Other

The case that magnetic sources interfere with each other and affect the detection of the magnetic source can be divided into two types. The first is due to the dipole moments of two magnetic dipoles differing significantly, and the second is due to the distance between two magnetic dipoles being close. To evaluate the ability of the VMGT${}_{2}$ angle to detect mutual interference magnetic sources, we set up two sets of simulation data corresponding to the above two cases, as shown in Table 6. The detection area is 10 m × 10 m. The height of the measurement plane is 0 m, and the interval between the measurement points is 0.25 m.

**Figure 7:**
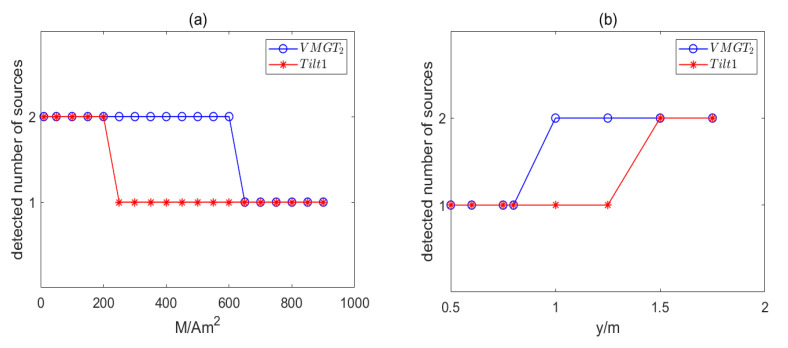
Detection results. (**a**) The first set of data; (**b**) the second set of data. The blue circle represent the number of magnetic sources detected based on the VMGT${}_{2}$ angle. The red asterisk indicates the number of magnetic sources detected based on Tilt1.

The first data set consists of dipole 1 and dipole 2. The moment of dipole 2 ranged from 10 $Am^{2}$ to 900 $Am^{2}$. It was used to verify the detection ability of the method in the face of the dipole moments of two magnetic dipoles differing significantly. We calculated the detection results of the first data set based on the VMGT${}_{2}$ angle and Tilt1, as shown in Figure 7a. The abscissa indicates the magnetic moment of dipole 2, and the ordinate indicates the number of detected magnetic sources. When the magnetic moment of dipole 2 is less than or equal to 600 $Am^{2}$, the method based on the VMGT${}_{2}$ angle correctly detected the two sources. The method based on Tilt1 correctly detected the two sources when the magnetic moment of dipole 2 was less than or equal to 200 $Am^{2}$. To evaluate the interference of dipole 2 to dipole 1, we calculated the ratio $r_{1}$ of the total magnetic field intensity (TMI) of dipole 2 at the measurement point directly above dipole 1 to the TMI of dipole 1 at that measurement point. We recorded it in Table 7. It can be seen that when the magnetic moment of dipole 2 was equal to 100 $Am^{2}$, its TMI at the measurement point directly above dipole 1 was already greater than the TMI of dipole 1 at the measurement point.

The second data set consisted of dipole 1 and dipole 3. The y-coordinate of dipole 3 was in the range of 0.5 m to 2.5 m. This data set was used to verify the detection ability of the method in the face of the distance between two magnetic dipoles being close. We calculated the detection results of the second data set based on the VMGT${}_{2}$ angle and Tilt1, as shown in Figure 7b. The method based on the VMGT${}_{2}$ angle correctly detected the two sources when the y-coordinate of dipole 3 was greater than or equal to 1 m. The method based on Tilt1 detected the number of sources correctly only when the y-coordinate of dipole 3 was greater than or equal to 1.5 m.

These experimental results show that the method based on the VMGT${}_{2}$ angle still has a certain detection capability when the field of a larger dipole moment completely covers the field of a smaller dipole moment. At the same time, the method based on the VMGT${}_{2}$ angle or based on the tilt angle has a limited detection capability for closer sources.

### 3.3. Field Demonstration

**Figure 8:**
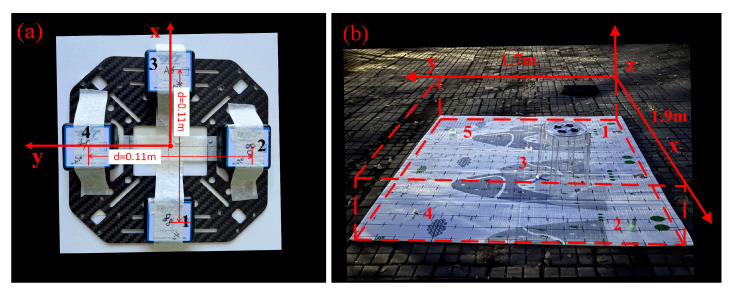
(**a**) Magnetic gradient tensor system. (**b**) Detection area.

To validate the applicability of our methods in engineering, we conducted a field demonstration in Harbin, China. We built a simple magnetic gradient tensor system for data acquisition based on the principle [24] of building a magnetic gradient tensor system with a cross structure. This magnetic gradient tensor system consists of four commercial three-axis fluxgate sensors placed on a carbon fibre cross-platform, as shown in Figure 8a. The fluxgate was a AK8963 3-axis magnetometer with 0.0667 mG/LSB resolution. Its measurement range is from −4912 μT to +4912 μT. The baseline distance between sensors was 0.11 m.

The measurement area was 1.9 m × 1.7 m, and the interval between the measurement points was 0.1 m (this scale is the equivalent reduction to some typical magnetic anomaly detection scenarios, such as searching for an underground unexploded ordnance, and searching for black boxes in aircraft wreckage, etc.). The magnetic gradient tensor system was placed 0.45 m above the ground. During the measurement process, the direction of the MGT system was kept constant, and the point-by-point acquisition was performed by moving the MGT system. We placed five magnetic sources with different magnetic moments at different locations and depths in the detection area, as shown in Figure 8b. These five magnetic sources were composed of one, three, two, two, and one identical magnet (Nd-Fe-B magnet with the size of *D*24 mm × *L*9 mm), respectively, and the magnetic inclination of magnetic sources 1, 2, and 4 were opposite to magnetic sources 3 and 5. Although the exact values of their magnetic moment magnitude and direction are unknown, we can know the positive and negative of the magnetic moment inclination angle I and the numerical relationship of the magnetic moment M based on the placement direction of the magnetic source and the number of magnets that make up the magnetic source. The location parameters, the sign of I, and the numerical relationship of M are recorded in Table 8.

**Figure 9:**
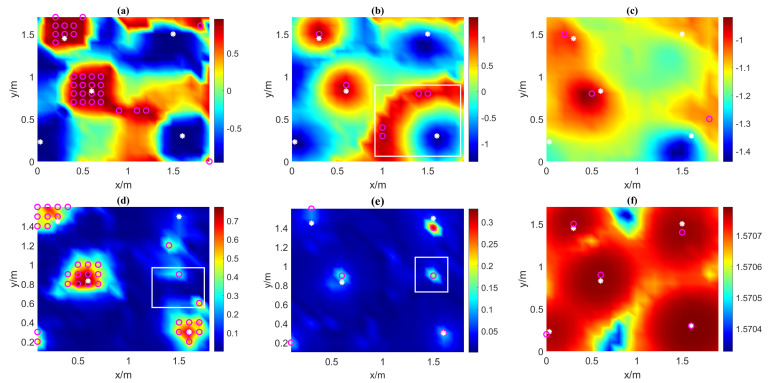
Calculated tilt angles and the VMGT${}_{2}$ angle. (**a**) Tilt1; (**b**) Tilt2; (**c**) Tilt3; (**d**) $T_{N}$; (**e**) $T_{C}$; (**f**) the VMGT${}_{2}$ angle. The white asterisks represent the real magnetic sources, the rose-red circles represent the detected magnetic sources, and the white rectangles represent the spurious magnetic anomaly regions.

We calculated the VMGT${}_{2}$ angle and the tilt angle on the measurement plane and plotted it in Figure 9. The white asterisks represents the real magnetic sources. The rose-red circles in Figure 9a,b,d,e are the sources obtained by the threshold method (threshold was 0.955, 1.33, 0.34, 0.089, respectively), and in Figure 9c,f are the sources obtained by the local maximum method. The white rectangles are the spurious magnetic anomaly region. Obviously, in Figure 9a–c, there are more abnormal regions due to the presence of magnetic sources with different positive and negative inclinations simultaneously, and there are also some outliers between different magnetic sources in Figure 9d,e, which affect the correctness of the result. The above two issues do not appear in Figure 9f, and the number of magnetic sources is correctly detected based on the $VMGT_{2}$ angle.

The final results optimized by the DE-LM algorithm were recorded in Table 8. The position estimation error of the magnetic source is $E_{p}=20$ mm. The inclinations’ positive and negative are consistent with the prearranged, and the magnitude of the magnetic moment corresponds to the number of magnets.

## 4. Conclusions

This paper proposes a new method for DLC of multiple dipole-like magnetic sources based on MGT data. Firstly, we propose the VMGT${}_{2}$ angle and use it to detect the number of magnetic sources in the area. Then, we use NSS to narrow the search space of magnetic source horizontal parameters. Finally, we use the DE-LM algorithm to optimize the parameters of magnetic sources to locate and identify magnetic sources. Through simulation experiments and a field demonstration, we verified that the method based on the VMGT${}_{2}$ angle can effectively solve the problem that the existing method based on the tilt angle cannot accurately estimate the number of magnetic sources when the detection area contains sources with positive and negative inclinations simultaneously. At the same time, we compared the convergence ability and convergence speed of the DE-LM algorithm, the PSO-LM algorithm, and the DE algorithm. The result shows that the DE-LM algorithm can improve the convergence speed, while ensuring the high-precision parameter solution in the DLC of multiple dipole-like magnetic sources. The experiment result shows that our method can correctly estimate the number of magnetic sources when the detection area contains sources with positive and negative inclinations simultaneously. For noise-free cases, the DE-LM algorithm converges to a high accuracy solution $(E_{p}<1$ mm, $E_{o}<10^{-3}\;Am^{2})$, and the convergence speed was improved by more than three times from the DE algorithm. For cases of 10% Gaussian random noises, the parameter optimization accuracy of the DE algorithm and DE-LM algorithm was similar, but the DE-LM algorithm’s convergence speed was 7.105 times faster than that of the DE algorithm. In the field demonstration, the position error of the magnetic source based on the DE-LM algorithm was $E_{p}=20$ mm. These results mean that the DE-LM algorithm can improve the convergence speed, while ensuring the high-precision parameter solution in the DLC of multiple dipole-like magnetic sources.

Although our proposed method has some ability to detect mutually interfering (due to the dipole moments of magnetic dipoles differing significantly or the distance between magnetic dipoles being close) magnetic sources, as the interference increases, it is difficult to correctly estimate the number of magnetic sources, which is a common problem in existing methods. In the future, we will study high-resolution methods for detecting mutually interfering magnetic sources.

## Figures and Tables

**Table 1:** Prearranged locations and moments of five magnetic dipoles.

Dipole	x/m	y/m	z/m	$I{/}^{\circ }$	$D{/}^{\circ }$	$M/{\mathrm{Am}}^{2}$
1	−6.1	−7.7	3.3	4	63	35
2	4.4	−8.9	1.8	−76	−148	10
3	−4.9	5.4	0.7	67	116	12
4	0.1	0.2	1.4	−45	−164	11
5	3.2	6.7	1.2	22	−39	15

**Table 2:** Approximate horizontal coordinates and search space for parameters of magnetic dipoles.

Approximate Horizontal Coordinates			Search Space					
**Dipole**	**x/m**	**y/m**	**x/m**	**y/m**	**z/m**	$I{/}^{\circ }$	$D{/}^{\circ }$	$M/{\mathrm{Am}}^{2}$
1	−6.50	−7.50	−7.00∼−6.00	−8.00∼−7.00	0∼50	−90∼90	−180∼180	0∼500
2	4.50	−8.75	4.00∼5.00	−9.25∼−8.25	0∼50	−90∼90	−180∼180	0∼500
3	−5.00	5.50	−5.50∼−4.50	5.00∼6.00	0∼50	−90∼90	−180∼180	0∼500
4	−0.25	0.25	−0.75∼0.25	−0.25∼0.75	0∼50	−90∼90	−180∼180	0∼500
5	3.25	7.00	2.75∼3.75	6.50∼7.50	0∼50	−90∼90	−180∼180	0∼500

**Table 3:** Estimated locations and moments of five magnetic dipoles.

Noise	Method	Dipole	x/m	y/m	z/m	$I{/}^{\circ }$	$D{/}^{\circ }$	$M/{\mathrm{Am}}^{2}$
0	PSO-LM [20]	1	−6.114 (0.014)	−7.726 (0.026)	3.215 (0.085)	5.037 (1.037)	62.793 (0.207)	31.768 (3.232)
		2	4.398 (0.002)	−8.896 (0.004)	1.801 (0.001)	−75.973 (0.027)	−146.060 (1.940)	10.028 (0.028)
		3	−4.900 (0)	5.400 (0)	0.700 (0)	66.981 (0.019)	115.987 (0.013)	12.002 (0.002)
		4	−83.369 (83.469)	−146.822 (392.978)	108.032 (466.632)	106.336 (−28.664)	77.565 (118.435)	−159.751 (170.751)
		5	3.200 (0)	6.700 (0)	1.200 (0)	21.965 (0.035)	−38.985 (0.015)	14.987 (0.013)
	DE [15]	1	−6.1 (0)	−7.7 (0)	3.3 (0)	4 (0)	63 (0)	35 (0)
		2	4.4 (0)	−8.9 (0)	1.8 (0)	−76 (0)	−148 (0)	10 (0)
		3	−4.9 (0)	5.4 (0)	0.7 (0)	67 (0)	116 (0)	12 (0)
		4	0.1 (0)	0.2 (0)	1.4 (0)	−45 (0)	−164 (0)	11 (0)
		5	3.2 (0)	6.7 (0)	1.2 (0)	22 (0)	−39 (0)	15 (0)
	DE-LM	1	−6.1 (0)	−7.7 (0)	3.3 (0)	4 (0)	63 (0)	35 (0)
		2	4.4 (0)	−8.9 (0)	1.8 (0)	−76 (0)	−148 (0)	10 (0)
		3	−4.9 (0)	5.4 (0)	0.7 (0)	67 (0)	116 (0)	12 (0)
		4	0.1 (0)	0.2 (0)	1.4 (0)	−45 (0)	−164 (0)	11 (0)
		5	3.2 (0)	6.7 (0)	1.2 (0)	22 (0)	−39 (0)	15 (0)
10%	DE [15]	1	−6.099 (0.001)	−7.706 (0.006)	3.333 (0.033)	4.283 (0.283)	62.929 (0.071)	35.205 (0.205)
		2	4.392 (0.008)	−8.891 (0.009)	1.800 (0)	−76.767 (0.767)	−145.469 (2.531)	9.892 (0.108)
		3	−4.900 (0)	5.401 (0.001)	0.701 (0.001)	66.735 (0.265)	115.647 (0.353)	12.101 (0.101)
		4	0.098 (0.002)	0.204 (0.004)	1.401 (0.001)	−44.821 (0.179)	−163.212 (0.788)	11.045 (0.045)
		5	3.200 (0)	6.699 (0.001)	1.203 (0.003)	21.946 (0.054)	−39.036 (0.036)	14.997 (0.003)
	DE-LM	1	−6.101 (0.001)	−7.695 (0.005)	3.312 (0.012)	3.974 (0.026)	63.049 (0.049)	35.334 (0.334)
		2	4.406 (0.006)	−8.903 (0.003)	1.796 (0.004)	−75.747 (0.253)	−149.487 (1.487)	9.931 (0.069)
		3	−4.905 (0.005)	5.404 (0.004)	0.702 (0.002)	66.146 (0.854)	116.969 (0.969)	12.075 (0.075)
		4	0.095 (0.005)	0.200 (0)	1.405 (0.005)	−44.912 (0.088)	−163.543 (0.457)	11.107 (0.107)
		5	3.197 (0.003)	6.693 (0.007)	1.209 (0.009)	561.970 (0.030)	−399.493 (0.493)	−15.196 (0.196)

The corresponding absolute errors are listed in parentheses.

**Table 4:** Prearranged parameters of 40 magnetic dipoles.

Dipole	x/m	y/m	z/m	$I{/}^{\circ }$	$D{/}^{\circ }$	$M/{\mathrm{Am}}^{2}$	Dipole	x/m	y/m	z/m	$I{/}^{\circ }$	$D{/}^{\circ }$	$M/{\mathrm{Am}}^{2}$
1	−13.20	3.30	0.60	16.00	−2.00	10.00	21	−6.1	16.80	0.70	86.00	128.00	9.00
2	−17.90	−16.90	0.70	−73.00	78.00	10.00	22	−24.3	21.40	0.70	37.00	−127.00	7.50
3	−15.30	−2.10	1.10	17.00	174.00	7.50	23	−22.3	−17.20	0.70	69.00	179.00	7.50
4	19.90	−7.20	0.90	46.00	−7.00	9.00	24	15.3	−2.20	0.80	12.00	44.00	8.00
5	23.10	20.10	1.40	72.00	−113.00	8.50	25	4.3	9.20	0.40	86.00	49.00	10.00
6	9.70	−15.60	0.70	45.00	155.00	10.00	26	−3.3	7.10	1.20	12.00	155.00	9.00
7	0.10	−14.10	0.80	−66.00	86.00	7.00	27	−8.4	7.20	0.90	85.00	−143.00	14.50
8	14.90	−10.10	0.90	42.00	69.00	11.00	28	11.3	16.10	1.30	−13.00	−105.00	10.00
9	−18.10	17.90	1.00	40.00	143.00	12.50	29	17.2	5.90	0.70	76.00	145.00	7.00
10	5.20	14.90	1.20	29.00	11.00	7.50	30	−9.1	−1.10	0.50	44.00	−130.00	10.00
11	−23.20	−3.30	1.40	58.00	−86.00	14.00	31	8.7	−1.30	1.00	48.00	−69.00	9.00
12	−20.60	−8.70	1.10	89.00	28.00	6.00	32	−15.6	−9.80	1.00	27.00	172.00	8.00
13	16.40	12.10	0.90	−85.00	78.00	8.50	33	−8.9	−17.20	0.60	89.00	−31.00	9.00
14	−21.10	3.30	0.90	23.00	−119.00	13.00	34	24.1	−20.40	0.60	61.00	−38.00	10.00
15	13.30	9.40	0.50	36.00	−93.00	8.00	35	20.1	5.60	1.10	13.00	78.00	12.00
16	18.10	−18.20	1.50	41.00	169.00	17.00	36	6.9	4.30	1.00	−46.00	−135.00	8.00
17	−0.20	0.10	0.90	24.00	−8.00	8.00	37	7.6	−9.10	1.40	33.00	−21.00	12.00
18	17.90	18.10	0.50	35.00	−65.00	15.00	38	−16.2	10.10	1.20	29.00	137.00	9.00
19	−7.90	−9.90	0.80	52.00	−88.00	7.00	39	1.1	−6.40	1.10	−2.00	−109.00	6.00
20	20.40	11.20	1.20	20.00	−144.00	9.50	40	−12.1	14.20	0.80	6.00	−160.00	9.00

**Table 5:** Average error of 40 magnetic dipoles parameters under different noise levels.

Noise	x${}_{\mathrm{error}}$/m	y${}_{\mathrm{error}}$/m	z${}_{\mathrm{error}}$/m	$I_{\mathrm{error}}{/}^{\circ }$	$D_{\mathrm{error}}{/}^{\circ }$	$M_{\mathrm{error}}/{\mathrm{Am}}^{2}$
0	$7.610\times 10^{-8}$	$5.140\times 10^{-8}$	$1.885\times 10^{-8}$	$8.701\times 10^{-6}$	$1.152\times 10^{-4}$	$6.115\times 10^{-8}$
10%	0.014	0.012	0.010	1.917	2.895	0.026

**Table 6:** The parameters of magnetic dipoles.

Dipole	x/m	y/m	z/m	$I{/}^{\circ }$	$D{/}^{\circ }$	$M/{\mathrm{Am}}^{2}$
1	0	0	1	60	45	10
2	0	1.75	2	60	45	10~900
3	0	0~2	1	60	45	10

**Table 7:** The ratio $r_{1}$ corresponding to different magnetic moments of dipole 2.

$M/{\mathrm{Am}}^{2}$	50	100	200	250	300	500	600	650	700
$r_{1}$	0.2703	0.5407	1.0814	1.3518	1.6221	2.7036	3.2443	3.5146	3.7850

**Table 8:** Prearranged locations, estimated locations, and moments of the magnetic sources.

	Magnetic Sources	x/m	y/m	z/m	$I{/}^{\circ }$	$D{/}^{\circ }$	$M/{\mathrm{Am}}^{2}$
Prearranged	1	0.030	0.230	0	+	/	$M_{1}$
	2	1.600	0.300	0.050	+	/	3$M_{1}$
	3	0.600	0.830	0.025	−	/	2$M_{1}$
	4	1.500	1.500	0.025	+	/	2$M_{1}$
	5	0.300	1.450	0.050	−	/	$M_{1}$
Estimated	1	0.032	0.231	−0.008	83.430	−25.230	2.957
	2	1.607	0.318	0.046	66.813	−75.560	9.496
	3	0.605	0.839	0.021	−75.274	−58.967	5.163
	4	1.507	1.518	0.024	22.005	−93.837	4.720
	5	0.337	1.432	0.050	−38.165	−70.468	2.486

$M_{1}$ is the moment of the identical magnet.

## Data Availability

Not applicable.
